# Observing Action Sequences Elicits Sequence-Specific Neural Representations in Frontoparietal Brain Regions

**DOI:** 10.1523/JNEUROSCI.1597-18.2018

**Published:** 2018-11-21

**Authors:** Dace Apšvalka, Emily S. Cross, Richard Ramsey

**Affiliations:** ^1^Social Brain in Action Laboratory, Wales Institute for Cognitive Neuroscience, School of Psychology, Bangor University, Wales, LL57 2AS,; ^2^MRC Cognition and Brain Sciences Unit, University of Cambridge, Cambridge, United Kingdom, CB2 7EF, and; ^3^Institute of Neuroscience and Psychology, School of Psychology, University of Glasgow, Glasgow, Scotland G12 8QB

**Keywords:** action observation, fMRI, MVPA, sequence learning

## Abstract

Learning new skills by watching others is important for social and motor development throughout the lifespan. Prior research has suggested that observational learning shares common substrates with physical practice at both cognitive and brain levels. In addition, neuroimaging studies have used multivariate analysis techniques to understand neural representations in a variety of domains, including vision, audition, memory, and action, but few studies have investigated neural plasticity in representational space. Therefore, although movement sequences can be learned by observing other people's actions, a largely unanswered question in neuroscience is how experience shapes the representational space of neural systems. Here, across a sample of male and female participants, we combined pretraining and posttraining fMRI sessions with 6 d of observational practice to determine whether the observation of action sequences elicits sequence-specific representations in human frontoparietal brain regions and the extent to which these representations become more distinct with observational practice. Our results showed that observed action sequences are modeled by distinct patterns of activity in frontoparietal cortex and that such representations largely generalize to very similar, but untrained, sequences. These findings advance our understanding of what is modeled during observational learning (sequence-specific information), as well as how it is modeled (reorganization of frontoparietal cortex is similar to that previously shown following physical practice). Therefore, on a more fine-grained neural level than demonstrated previously, our findings reveal how the representational structure of frontoparietal cortex maps visual information onto motor circuits in order to enhance motor performance.

**SIGNIFICANCE STATEMENT** Learning by watching others is a cornerstone in the development of expertise and skilled behavior. However, it remains unclear how visual signals are mapped onto motor circuits for such learning to occur. Here, we show that observed action sequences are modeled by distinct patterns of activity in frontoparietal cortex and that such representations largely generalize to very similar, but untrained, sequences. These findings advance our understanding of what is modeled during observational learning (sequence-specific information), as well as how it is modeled (reorganization of frontoparietal cortex is similar to that previously shown following physical practice). More generally, these findings demonstrate how motor circuit involvement in the perception of action sequences shows high fidelity to prior work, which focused on physical performance of action sequences.

## Introduction

From learning to use chopsticks to dancing the lead role in “Swan Lake,” humans display a remarkable ability to learn complex new motor skills by watching others perform these actions. However, it remains unclear how visual signals are mapped onto motor circuits for such learning to occur. Indeed, our understanding of how action representations develop during motor learning through physical compared with observational practice remains in its infancy (Frey and Gerry, 2006; Hodges et al., 2007; Vogt and Thomaschke, 2007; McGregor et al., 2016; Ostry and Gribble, 2016). Here, we advance understanding of observational learning by using fMRI to test the idea that observational learning of action sequences leads to distinctive patterns of activity in sensorimotor cortices in a manner similar to that reported following physical practice (Wiestler and Diedrichsen, 2013).

Common brain regions have been shown to underpin motor learning following physical and observational experience (Cross et al., 2009; Kirsch and Cross, 2015; Ostry and Gribble, 2016). For example, if the motor system is engaged in another task (Mattar and Gribble, 2005) or if sensorimotor systems are disrupted through noninvasive stimulation (Brown et al., 2009; McGregor et al., 2016), observational learning is reduced. Further, neuroimaging studies have demonstrated that frontoparietal cortex shows similar changes in magnitude and connectivity when learning through physical and observational practice (Vogt et al., 2007; Cross et al., 2009; van der Helden et al., 2010; Higuchi et al., 2012; Sakreida et al., 2018). Although these studies demonstrate that sensorimotor cortices are involved in learning motor skills by observation, it remains unclear how visual signals are mapped onto motor circuits for learning to occur.

Compared with action observation and visual training, considerably more research has investigated neural representations underpinning action execution and physical training (Kelly and Garavan, 2005; Dayan and Cohen, 2011; Penhune and Steele, 2012; Hardwick et al., 2013; Diedrichsen and Kornysheva, 2015). fMRI studies have shown both increases and decreases in frontoparietal cortex engagement following motor learning, with increases argued to reflect additional recruitment of cortical tissue and decreases suggestive of more efficient neural function (Steele and Penhune, 2010; Dayan and Cohen, 2011; Gardner et al., 2017). However, because conventional fMRI analyses average activity across voxels, they are insensitive to a richness of information that is represented by the pattern of activity across voxels (Kriegeskorte et al., 2008). Sidestepping the issue of averaging across voxels, Wiestler and Diedrichsen (2013) used a motor learning paradigm in combination with multivoxel pattern analysis (MVPA) to identify how patterns of activity across voxels relate to mental content independently of average activity (Norman et al., 2006; Kriegeskorte et al., 2008). Wiestler and Diedrichsen (2013) showed that execution of kinematically matched key press sequences was associated with sequence-specific patterns of activity in multiple frontoparietal brain areas. Moreover, physically practicing sequences led to reduced activity on average and more distinctive patterns of activity in frontoparietal brain areas, implying a more distinct neural representation of learned sequences that enables faster execution (Wiestler and Diedrichsen, 2013).

To date, MVPA has been used to understand neural representations in a variety of domains, including vision, audition, memory, and action, but few studies have investigated neural plasticity in representational space (Kriegeskorte and Kievit, 2013). Therefore, although movement sequences can be learned by observing other people's actions (Blandin et al., 1999; Bird et al., 2005; Hodges et al., 2007; Vogt et al., 2007; Boutin et al., 2010), a largely unanswered question in neuroscience is how experience shapes the representational space of neural systems (Kriegeskorte and Kievit, 2013). To address this question, here we test the extent to which observation of action sequences elicits sequence-specific representations in frontoparietal brain regions and the extent to which these representations become more pronounced with observational practice. If observed sequences are mapped onto sensorimotor circuits in a similar manner to physical practice (Wiestler and Diedrichsen, 2013), we would expect sequence-specific patterns of activity to emerge within sensorimotor cortices following observational training.

## Materials and Methods

### 

#### Participants

Eighteen right-handed (based on self-report) volunteers from the Bangor University student community participated in the study. Two participants were not included in the final sample: a pilot participant who did not have the same testing parameters and a participant who made excessive head movements during one of the scanning sessions (>4 mm). The final sample comprised 16 participants (8 males and 8 females), 20–40 years old (M = 24.31 years, SD = 5.06). All participants had normal or corrected-to-normal vision and no history of neurological disorders. Participants gave their written informed consent and were paid £45 for their participation. All procedures were approved by the Ethics Committee of the School of Psychology at Bangor University and UK Ministry of Defense Research Ethics Committee (protocol 524/MODREC/14).

#### Stimuli

A key press sequence learning paradigm was implemented based on the task used by Wiestler and Diedrichsen (2013). A standard black QWERTY computer keyboard was used with the “Q,” “3,” “4,” “5,” and “Y” keys covered with red tape and all surrounding keys removed. In pretraining and posttraining sessions, participants were required to press the red keys with the five fingers of their left hand in a specified order. During the observational training and fMRI sessions, participants watched videos of the experimenter performing the key press task. For the video recordings, a similar keyboard was used, with the only difference that the sides of the five keys were covered in yellow to improve the visibility of the key being pressed. Stimuli presentation and response recordings were performed using MATLAB version 8.3.0 (The MathWorks) and Psychophysics Toolbox 3.0.12 (Brainard, 1997).

##### Key press sequences.

The same set of 12 five-element key press sequences was used as previously by Wiestler and Diedrichsen (2013). Each sequence required the five fingers of the left hand to be pressed once in a sequential order, with each of the 12 sequences featuring a different order with no more than three adjacent finger presses in a row. All sequences were matched for difficulty based on a pilot experiment (Wiestler and Diedrichsen, 2013). For each participant, from the set of 12 sequences, four sequences were randomly allocated to the trained condition and four other sequences were allocated to the untrained condition. The remaining four sequences remained unused.

##### Videos.

**Figure 1.**
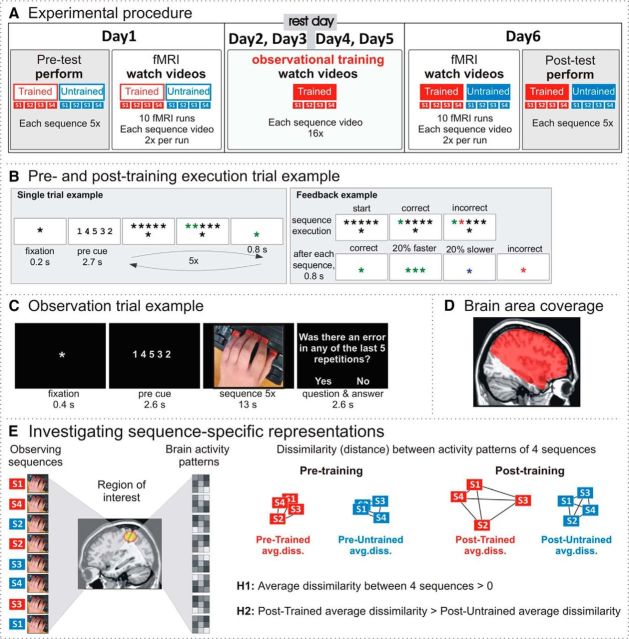
Experimental paradigm. ***A***, Experimental procedure. The experiment involved pretest and posttest, separated by 4 training days and 2 scanning (fMRI) sessions. In the pretest and posttest, participants performed eight key press sequences (four of them to be trained, the other four untrained). In the scanning sessions, participants watched videos of a hand performing the same eight sequences. In the training sessions, participants watched videos of a hand performing four of the eight sequences. ***B***, Execution trial example. A cued sequence had to be memorized and then executed five times while receiving performance feedback. ***C***, Observation trial example. A sequence cue was followed by a video showing a hand executing the sequence five times, either correctly or incorrectly. Occasionally a question was asked whether there was an error in any of the five repetitions and a response had to be made. ***D***, Brain area coverage for fMRI analysis focused on premotor and parietal brain regions and did not include the cerebellum, occipital lobes, or inferior temporal lobes. ***E***, During pretraining and posttraining fMRI sessions, participants watched videos of eight different sequence executions: four sequences belonged to trained and four others to untrained conditions. Within each condition and each fMRI session, we measured the dissimilarity between each pair of the four sequences (six pairs) and obtained the average dissimilarity estimate (LDC) between the four sequences. The dissimilarity measures were used to investigate our main hypotheses: (1) that action observation evokes movement-sequence-specific brain activity patterns (the average dissimilarity between four sequences is above zero) and (2) that the activity patterns become more distinct following observational practice (the average dissimilarity between four trained sequences is higher than between four untrained sequences).

For observational training and both scanning sessions, 13 s videos were created showing the experimenter's left hand from a first-person perspective slightly tilted to the right (Fig. 1*C*; see Stimuli, https://osf.io/jz4nk/). Each video showed the experimenter executing one sequence five times with naturally varying breaks between each sequence repetition to ensure a more authentic presentation of the performance. For the same reason, for each sequence, five different video versions were recorded to allow closer to natural performance variation of the same sequence. An additional video version for each sequence was created in which one of the five sequence executions was incorrect. This resulted in 72 videos in total.

Sequences were executed at an intermediate performance level, which was determined by behavioral pilot test results in which the average time to complete a correct sequence execution was 2.29 s (*n* = 17, M = 2.29 s, SE = 0.14). Each original video showing five repetitions of the same sequence was slightly speeded up or slowed down (±10%) to make it exactly 13 s long. Therefore, the authenticity of movement performance was somewhat reduced, but the relative variability within the video remained intact. The average single sequence execution in the videos was 2.3 s. The videos were presented on a computer monitor in full color on a black background. The frame rate was 29 frames/s with the resolution of 600 × 526 pixels showing approximately natural hand size.

#### Procedure

##### Overview.

Participants underwent 6 testing days over a 7 d period (Fig. 1*A*). On the first day of testing, participants received task instructions and completed three single-sequence execution trials to ensure that each participant understood the task. The familiarization procedure was followed by a pretraining session, which was immediately followed by the first scanning session. Participants returned to the laboratory for the next 2 consecutive days for observational training sessions, which were followed by a day off. After the rest day, participants returned to the laboratory for 2 more consecutive days of observational training sessions. The final day (day 6) started with the second scanning session immediately followed by a posttraining session. Each session is described in more detail below.

##### Pretraining and posttraining sessions.

In the pretraining and posttraining sessions, participants performed four trained and four untrained sequence execution trials in a random order with their left hand. Each trial consisted of five repetitions of the same sequence (Fig. 1*B*). All trial-related information was presented centrally at the bottom of the screen against a gray background. A trial started with a black fixation cross (0.2 s), followed by the sequence cue presented as five digits (2.7 s) that indicated from right to left which key to press: “1,” the right-most key pressed with the thumb, and “5,” the left-most key pressed with the little finger. After the cue, the digits were replaced by the fixation cross and five black asterisks above it. This served as a “go” signal to execute the memorized sequence five times as quickly and accurately as possible. If the correct key was pressed, then the corresponding asterisk on the screen turned green; if a wrong key was pressed, the asterisk turned red.

After executing a single sequence, the central fixation cross changed color to provide feedback on the performance (0.8 s): green was a correct sequence execution; red was an incorrect sequence execution; blue was correct but executed 20% slower than the median execution time in the previous trials; and three green asterisks was correct and executed 20% faster than the median execution time in the previous trials. After this short feedback, all asterisks turned black, signaling the start of the next execution trial. After five executions of the same sequence, the trial ended and the next sequence was cued.

##### Observational training sessions.

In the observational training sessions, participants watched videos of the four trained sequence executions. Participants were instructed to watch the videos and to pay close attention to whether the sequences were performed correctly. Occasionally, they would be asked whether the performer in the video made an error in any of the five repetitions: the error question. They would respond by pressing the “b” key (marked red) on a keyboard for “yes” and the “m” key (marked blue) for “no.” This task was included to ensure that participants paid close attention to the videos. Participants were also informed that they would need to perform the watched sequences again at the end of the experiment.

All trial-related information was presented in the middle of the screen against a black background with a light gray font (Fig. 1*C*). A trial started with a fixation cross (0.4 s), followed by the sequence cue presented as 5 digits (2.6 s), followed by the sequence video (13 s). After some of the trials, the error question was asked and participants had 2.6 s to respond.

A training session was divided into four blocks separated by a rest period. Within each block, 20 videos were presented in a random order. Each of the four training sequence videos was shown four times (randomly choosing one of the five video versions for each sequence, described in the “Videos” section above). There was also one “error video” for each sequence (where at least one of the five repetitions of the sequence execution was incorrect). The error question appeared randomly five to seven times per block. At the end of each block, participants received feedback on how accurately they spotted the incorrect sequence executions. The whole training session lasted ∼25 min and participants saw a correct execution of each sequence at least 80 times (four blocks, four distinct sequence videos per block, five repetitions of a single sequence per video, plus some correct repetitions in the error video).

##### Scanning sessions.

During identical pretraining (day 1) and posttraining (day 6) fMRI sessions, participants observed the four trained and four untrained sequence videos in a random order. The observation trials were structured in the same way as in the observational training sessions (Fig. 1*C*). In each scanning session, participants completed 10 functional runs. Each functional run comprised 17 videos presented in a random order: eight sequence videos presented twice each and one error video. Each video showed five repetitions of one sequence. Therefore, during each scanning session, participants saw a correct execution of each sequence at least 100 times (10 functional runs, two videos per sequence per run, five repetitions of a single sequence per video, plus some correct repetitions in the error video).

In keeping with the observational training sessions, participants were instructed to watch whether all sequences were correctly executed and to answer the error question when asked. The error question was asked twice within a run, always after the error video and randomly after one of the correct videos. Each run also had five rest phases, one at the beginning of the run and four randomly interspersed, but never twice in a row. The rest phase was 13 s long and showed a fixation cross in the middle of the screen. Each run lasted ∼6 min (2.6 s per whole-volume acquisition, with 138 acquisitions per run).

Stimuli were presented onto a screen located behind the MRI scanner and displayed to the participant via a mirror positioned above participants' eyes. Responses to the error questions were recorded using a scanner-safe fiber optic four-button response pad (Current Designs) connected to the stimulus PC.

#### Scan acquisition

MRI data were acquired using a 3 tesla Achieva MRI scanner (Philips Health Care) fitted with a sensitivity-encoded (SENSE) 32-channel phased-array head coil.

##### Functional scans.

Both scanning sessions consisted of 10 functional runs of the blood-oxygenation-level-dependent (BOLD) signal acquisitions (Ogawa et al., 1992), with two dummy scans followed by 136 scans per run. Volumes were collected using a T2*-weighted single shot gradient echoplanar imaging sequence with the following parameters: TE = 30 ms, TR = 2.6 s, flip angle = 90°, 41 ascending slices with 2.3 mm thickness, 0.15 mm gap, and 2 × 2 mm^2^ in-plane resolution (matrix size 96 × 96). The slice acquisition was focused on premotor and parietal brain regions, so the group average brain area coverage did not include the cerebellum or all of the occipital or inferior temporal lobes (Fig. 1*D*).

##### Anatomical scan.

The last scanning session (day 6) ended with a high-resolution whole-brain 3D anatomical scan acquired as a T1-weighted image (MP-RAGE, TE = 3.5 ms, TR = 12 ms, voxel resolution = 1 mm^3^, slice thickness = 2 mm, flip angle = 8°), which was used as an anatomical reference for each participant.

#### Experimental design and statistical analysis

##### Overview of analysis strategy.

The general analysis strategy was motivated by our main research question, which focused on understanding how changes in the pattern of activity in frontoparietal cortex supports sequence-specific representations following observational learning. More specifically, we measured the extent to which individual observed action sequences are represented by patterns of activity in frontoparietal cortex, as well as the extent that these sequence-specific representations are dissociable in a training-specific manner (i.e., trained > untrained). In addition, we focused our pattern analyses on specific regions of interest (ROIs) that, as measured by average activity across voxels, showed sensitivity to observational learning during the posttraining scan session (Sakreida et al., 2018). By focusing our pattern analyses on regions that satisfy functional criteria associated with observational learning, we ensure that inferences drawn regarding representational-level and sequence-specific effects are in brain regions that are sensitive to observational learning.

The analyses performed within these ROIs closely follow analyses reported in prior physical training studies that have employed a sequence learning task (Wiestler and Diedrichsen, 2013; Wiestler et al., 2014). In terms of behavioral effects following sequence learning, Wiestler and colleagues (2013, 2014), reported skill learning that generalized across all sequences (significant pretraining to posttraining performance improvement of both trained and untrained sequences) and training-specific sequence learning (greater posttraining performance for trained than untrained sequences).

In addition, in frontoparietal brain regions, measures of average activity and MVPA showed evidence for generalized skill learning and training-specific effects (Wiestler and Diedrichsen, 2013). Here, we performed similar analyses of behavioral and brain data to test the extent to which observational training effects generalize across trained and untrained sequences and whether these effects dissociate between trained compared with untrained sequences. To do so, we first assessed sequence-specific learning for trained and untrained sequences separately. That is, we assessed the extent to which distinctive patterns of activity for observed action sequences (regardless of training condition) are identifiable within task-defined regions of the frontoparietal cortex. This first analysis is an important extension to prior sequence-learning action observation studies that used univariate measures (Frey and Gerry, 2006; Sakreida et al., 2018) because univariate measures are unable to distinguish between the neural representation of individual sequences. Indeed, univariate measures can distinguish between a collection of trained and untrained sequences, but the coarseness of univariate measures does not allow individual sequences to be distinguished. Therefore, the inference drawn from univariate measures may suggest that observed sequences are represented in frontoparietal cortex in a relatively coarse manner (e.g., five key presses in any order). However, by analyzing the pattern of activity across voxels, we are able to ask questions about the structure of observed action sequence representations on a more fine-grained level than previously possible. By doing so, theories can be developed and tested that have an enhanced level of specificity and suggest that observed actions are discriminable at an individual sequence level in frontoparietal cortex in a similar fashion to physically performed actions.

Second, to test the extent to which sequence-specific patterns of activity in frontoparietal cortex dissociate between sequences in a training-specific manner, we assessed differences between representations of trained and untrained sequences during the posttraining scan session. Before training, because all sequences are equally unfamiliar, there is no theoretical reason for any systematic difference between to-be-trained and to-remain-untrained sequences on average across the sample. This said, it is unlikely that every participant's data will reflect zero difference between to-be-trained and to-remain-untrained sequences and, as a consequence, we might expect some degree of nonzero variability across individual participants. To correct for possible pretraining differences between trained and untrained sequences, we followed the approach by Wiestler and Diedrichsen (2013) and calculated a linear regression between the pretraining difference (predictor) and the posttraining difference (outcome). The intercept of the regression line was used as a measure of the posttraining difference between trained and untrained conditions, correcting for possible pretraining differences. By including any small idiosyncratic pretraining differences in our regression model, we took into account of any nuisance impact that such differences may have on our primary analysis and thus focus on testing more directly our primary research questions. Therefore, by accounting for the initial training differences, we were able to remove a source of noise, increase the power, and, most importantly, maximize the measurement of our effects of interest. The linear regression approach was used in all subsequent behavioral and brain imaging analyses (univariate and MVPA) when comparing trained and untrained conditions after training.

Finally, to complement these ROI-based pattern analyses, we also performed a whole-volume searchlight analysis. Because sequence-specific representations of observed action sequences have not been investigated before, the whole-volume searchlight analysis enables us to characterize our main research questions beyond our ROIs. It is important to note that the whole-volume analysis did not cover the whole brain, but was instead restricted to frontoparietal cortex (Fig. 1*D*). Therefore, the cerebellum and parts of occipitotemporal cortex were not covered and these brain regions may be of interest for future investigations. This partial brain coverage was selected to maximize signal over our *a priori* ROIs in frontoparietal cortex.

##### Behavioral performance.

Participants' physical performance was assessed pretraining and posttraining, measuring the average sequence initiation time, execution time, and error rate of the four trained (to-be-trained) and the four untrained sequences. The sequence initiation time was measured as the duration between the “go” signal and the first key press. The sequence execution time was measured as the duration between the first and fifth key presses. The error rate was measured as the percentage of incorrect sequence executions. Incorrectly executed trials were excluded from further analysis. Attention to the task during the observational training and scanning sessions was assessed as a percentage of accurate responses to questions on error trials.

##### Imaging data.

Imaging data were analyzed using SPM12 (Wellcome Trust Centre for Neuroimaging, London), and custom-written MATLAB scripts. To correct for head motion, all images from a single scanning session (10 × 136 volumes) were spatially realigned to the mean functional image and slice-time corrected. The anatomical T1-wighted image was coregistered to the session-mean functional image and segmented to obtain parameters for spatial normalization. The time series of each voxel were high-pass filtered with a cutoff frequency of 1/52 Hz to remove low-frequency trends and modeled for temporal autocorrelation across scans with an AR(1) process.

For the voxelwise univariate analysis, normalization parameters from the segmentation step were used to normalize preprocessed functional images to the Montreal Neurological Institute (MNI) template brain with a resolution of 2 mm^3^. Normalized images were then spatially smoothed with a 3D Gaussian kernel of 8 mm full-width-half-maximum (FWHM). MVPA was performed without normalization and smoothing to preserve high spatial resolution.

All statistical maps were thresholded at a single-voxel level with a significance value of *p* < 0.001 and a minimum cluster size of 10 voxels (Friston et al., 1994). We based our approach on the most commonly used cluster-extent threshold (Carp, 2012), which has previously been shown to provide a desirable compromise between type I and type II error rates (Lieberman and Cunningham, 2009). To control for false-positive results, only brain regions reaching cluster-level familywise error (FWE) corrected significance at *p* < 0.05 are reported. For anatomical and cytoarchitectonic localization, we used SPM Anatomy toolbox version 2.0 (Eickhoff et al., 2005).

##### Univariate analysis.

The univariate analyses were designed to achieve two main objectives: (1) identify brain regions engaged in action observation and (2) identify brain regions sensitive to observational practice. Normalized and smoothed data were analyzed using a general linear model (GLM). A random-effects model was implemented at two levels. At the first level, single participant data were modeled by a single design matrix for all runs within each session. The design matrix contained six regressors of the following events: trained videos, untrained videos, an error video, error questions/responses, trained cues, and untrained cues. Trained and untrained video regressors (further named trained and untrained) represented the 13 s video duration (showing five repetitions of a single sequence execution). All regressors were modeled as boxcar functions convolved with a hemodynamic response function (HRF). The rest periods formed an implicit baseline.

To identify brain regions engaged in action observation, only data from the pretraining scanning session were used. Both action observation conditions of the pretraining session were taken together and contrasted with the implicit baseline (pretrained ∪ pre-untrained > implicit baseline). The first level whole-volume contrast maps were then entered into a second-level one-sample *t* test analysis to obtain group average results of brain areas engaged when watching key press sequences, in general, pretraining.

To identify brain regions sensitive to observational practice, the linear regression approach was used, as described above in the “Overview of the analysis strategy” section. Specifically, the pretraining difference between the estimated β weights of the trained and untrained conditions within each of the 10 pretraining functional runs was used as a predictor variable. The posttraining difference between the trained and untrained conditions within each of the 10 posttraining functional runs was used as an outcome variable. The intercept of the regression line was used as a measure of the posttraining difference between the trained and untrained conditions, correcting for possible pretraining differences. The linear regression was performed at the first level in a voxelwise manner across the whole volume and produced the intercept maps for each subject. These first-level whole-volume maps were then entered into a second-level one-sample *t* test analysis to obtain group average results of brain areas sensitive to observational practice.

##### ROI definition.

Based on univariate data, peak voxels from significant clusters showing the posttraining difference between Trained and Untrained conditions (independent of the direction) were used to create ROIs for MVPA. We note that our analysis approach is not circular (Kriegeskorte et al., 2009) because the univariate analysis of posttraining differences is statistically independent to all subsequent analyses.

**Figure 2.**
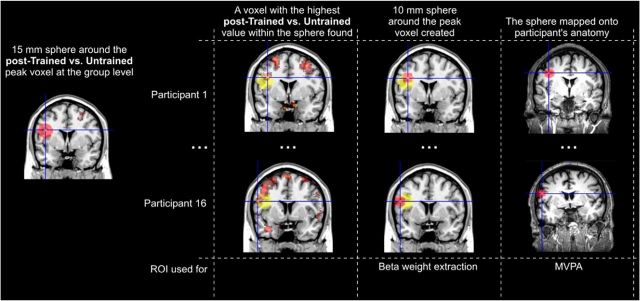
ROI definition procedure. The peak voxels of significant clusters showing the training-related changes in magnitude of brain activity were selected for ROI-based MVPA analyses. First, 15-mm-radius spheres were created in the MNI space centered on the group level voxels with the highest *t*-value of the posttraining difference between trained and untrained conditions (independent of the direction). Second, at a participant level, each individual's peak voxels were identified within the group level 15-mm-radius spheres. Third, 10-mm-radius spheres centered on the identified individuals' peak voxels were created for β-weight extraction. Fourth, the 10-mm-radius spheres were mapped from the MNI space onto individual subject anatomies for MVPA analysis.

More specifically, the ROIs were defined for each participant as follows (Fig. 2). First, 15-mm-radius spheres centered on the group level voxels with the highest *t*-value of the posttraining difference were created in MNI space (these ROIs are available at http://neurovault.org/collections/1892/). Second, at an individual participant level, voxels with the highest posttraining difference value within the 15-mm-radius spheres were selected as the individual's peak voxels. This approach was taken to account for anatomical and functional variability in the areas responsive to the task across participants. Third, 10-mm-radius spheres centered on the individuals' identified peak voxels were created for β-weight extraction to visualize the response. Fourth, the 10-mm-radius spheres were mapped from the MNI space onto individual subject anatomies for MVPA analysis. Any voxels covered by ROIs that extended outside the brain were not included in further analyses.

#### MVPA

##### ROI approach.

MVPA was implemented to achieve two main objectives: (1) identify brain regions associated with sequence-specific representations through action observation and (2) identify the extent to which patterns of activity become more sequence specific following observational training of action sequences. To test whether the observation of action sequences is associated with sequence-specific representations in frontoparietal cortex, we used MVPA to analyze brain activity patterns that emerge when watching the four sequences within each condition (trained and untrained). Consistent with the previous physical training study (Wiestler and Diedrichsen, 2013), we first examined sequence-specific patterns within each condition separately to determine whether neural representations in frontoparietal cortices distinguish between observed key press sequences in general. Second, we then compared the results across training conditions to determine whether the patterns of activity in frontoparietal cortex become more distinct for trained compared with untrained sequences (Fig. 1*E*).

The dissimilarity between activity patterns was measured using cross-validated Mahalanobis distance (Diedrichsen et al., 2016), which is closely related to linear discriminant analysis (LDA) and therefore termed linear discriminant contrast (LDC). In a recent study, LDC proved to be the most reliable MVPA measure, outperforming other more popular measures, such as pattern classification (LDA and support vector machine) and Pearson correlation (Walther et al., 2016).

LDC is a continuous dissimilarity measure, which includes multivariate noise normalization (prewhitening), cross-validation, and does not depend on baseline activity. Similar to LDA, LDC compares two conditions using a linear discriminant that has been estimated with independent data. However, instead of a binary decision, which is then converted into classification accuracy, LDC computes the mean difference between the two conditions measured along the linear discriminant. Cross-validation ensures that the measured dissimilarities between conditions are not due to noise in the data that makes conditions appear to differ by chance, but instead it ensures that dissimilarity measures represent a true difference with a meaningful zero point (Diedrichsen et al., 2016; Walther et al., 2016). If a brain region differentiates between the two types of stimuli (or two conditions), the average cross-validated dissimilarity measure of the activity patterns would be above zero.

Here, the LDC analysis was implemented using the RSA Toolbox (Nili et al., 2014) and custom-written MATLAB scripts. To obtain activity patterns for LDC analysis, a first-level GLM was estimated for each participant using the spatially realigned and slice-time-corrected images without normalization and smoothing. For the pretraining and posttraining data separately, a unique regressor for each of the eight sequences (four trained, four untrained) within each of the 10 runs was modeled as a boxcar function and convolved with an HRF. Each regressor averaged the brain activity across the two occurrences of the 13 s videos of each sequence within each run.

The LDC analysis of the activity patterns across sequences was performed for each condition (trained and untrained) and each participant separately. The estimated β weights of the voxels in each region (ROI or searchlight) were extracted and prewhitened to construct noise normalized activity patterns for each sequence within each run (Diedrichsen et al., 2016; Walther et al., 2016). Therefore, the input data for the LDC analysis consisted of 4 × 10 (4 sequences, 10 runs) activation estimates for a set of 160 neighboring voxels within each ROI. Leave-one-run-out cross-validated LDC analysis was performed and dissimilarity estimates averaged across the 10 possible cross-validation folds.

For each training condition and ROI separately, we compared patterns of activity between all four observed sequences to each other. This produced a total of six comparisons. For each comparison, we calculated the dissimilarity in patterns of activity as measured by the LDC. If the two patterns were dissimilar, the LDC value would be above zero, with greater dissimilarity producing a higher LDC value (Diedrichsen et al., 2016; Walther et al., 2016). In other words, if patterns of activity between two sequences were perfectly correlated, there would be zero dissimilarity. The resulting six dissimilarity scores were averaged to obtain the average dissimilarity estimate between the four sequences. An above-zero dissimilarity estimate indicates that the examined region (ROI or searchlight) has a pattern of activity that represents sequence-specific information.

For MVPA ROI analyses, we used a random subspace approach to increase the reliability of LDC measures (Diedrichsen et al., 2013). To do so, for each ROI separately, subsets of 160 voxels were randomly selected 1000 times. LDC analysis was performed on each subset and dissimilarity estimates from all 1000 subsets were averaged to obtain the final LDC measure for each ROI and each condition: LDC pretrained, LDC pre-untrained, LDC posttrained, and LDC post-untrained. Results were then submitted for statistical analyses.

First, we estimated the condition-average sequence-specific coding pretraining and posttraining separately. To do so, for the pretraining and posttraining scanning data separately, we averaged the trained and untrained LDC values and tested them against zero using one-tailed *t* tests. An above zero value would indicate that patterns of activity are distinct between sequences. Next, we assessed the posttraining difference (intercept) between the training conditions (trained > untrained), correcting for the possible pretraining differences (as described previously). All tests were Bonferroni corrected for the number of ROIs.

##### Searchlight approach.

In an exploratory whole-volume analysis, we performed a surface-based searchlight analysis (Oosterhof et al., 2011) to identify brain regions coding sequence-specific information across the whole cortical surface that was imaged (Kriegeskorte et al., 2006). Cortical surfaces were reconstructed from individual T1-weighted images using FreeSurfer (Dale et al., 1999). Around each surface node, spheres of searchlights were defined and all voxels between pial and white–gray matter surface were selected for analysis. The radius of each sphere was adjusted such that each searchlight contained exactly 160 voxels. The average searchlight radius was 10.37 mm.

For each searchlight, LDC analysis was performed for the four sequences within each condition as described in the MVPA ROI analysis section above. The dissimilarity estimate of each searchlight was assigned to the central voxel, constructing a surface map of dissimilarity estimates. The acquired individual subject maps (LDC pretrained, LDC pre-untrained, LDC posttrained, and LDC post-untrained) were then normalized to the MNI template with a resolution of 2 mm^3^ and spatially smoothed with a 3D Gaussian kernel of 4 mm FWHM.

The normalized and smoothed maps were then entered into a second-level random-effect analysis to obtain group average results of brain areas that code sequence-specific information when watching sequences pretraining and posttraining (one-sample *t* tests against zero of LDC pretrained ∪ LDC pre-untrained and of LDC posttrained ∪ LDC post-untrained). We also calculated the posttraining difference between the trained and untrained conditions, correcting for possible pretraining differences, using the linear regression approach as described previously.

In addition, following the Wiestler and Diedrichsen (2013) approach, we also inspected the sequence-specific representations globally, averaging over all involved cortical regions. Specifically, for each participant, we created a mask of cortical areas where the LDC value was above zero for any of the four conditions. Within this mask, for each condition separately, we calculated the average LDC value and the total area where the LDC value was above zero. Next, individual participant LDC and total area values were entered into the regression analyses to compare the posttraining difference between the trained and untrained conditions, correcting for possible pretraining differences (as described previously).

#### Reported confidence intervals and effect sizes

All sample means are reported with their 95% confidence intervals in square brackets. Confidence intervals for two-tailed tests were calculated as SE * 2.13, whereas confidence intervals for one-sided tests were calculated as SE * 1.74 for df 15 (Cumming, 2012). For paired comparisons, within-subject confidence intervals and effect sizes were used (Cousineau, 2005; Lakens, 2013).

#### Data sharing

Stimuli, data, and code for this study are freely available at https://osf.io/jz4nk/. In addition, we also performed an exploratory functional connectivity analysis using psychophysiological interaction (PPI) analyses (see https://osf.io/jz4nk/, “PPI_analysis”). Unthresholded fMRI maps, LDC maps, and group ROIs are uploaded at http://neurovault.org/collections/1892/.

## Results

### Behavioral data

**Figure 3.**
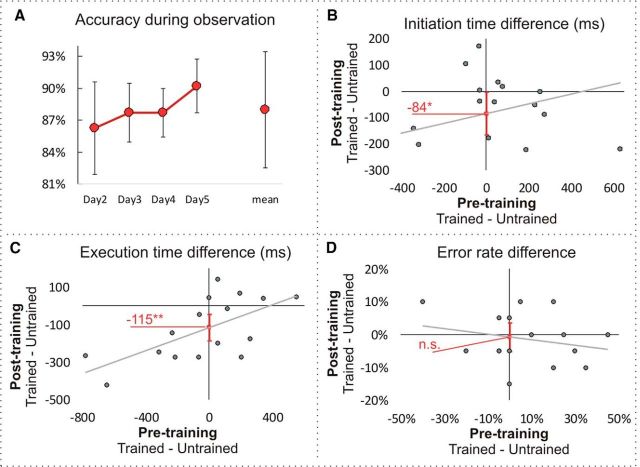
Behavioral results. ***A***, Group-averaged accuracy in response to the error question during observational training. Error bars represent within-subject 95% confidence interval. ***B***–***D***, Pretraining and posttraining difference in initiation time, execution time, and error rate between trained and untrained sequences. The training effect was measured as the intercept of the regression line between the pretraining difference (predictor) and the posttraining difference (outcome). The intercept represents the predicted posttraining difference if the pretraining difference is zero. This method reduces the noise of unwanted differences in the difficulty of trained and untrained sequences and thus allows a more accurate measurement of the training effect. Error bars represent 95% confidence interval of the intercept. *p < 0.05, **p < 0.01, n.s., Nonsignificant at *p* < 0.05.

We first assessed the extent to which participants were paying attention to the videos during observational training and scanning sessions by analyzing accuracy of performance on identifying error videos. The average accuracy across the 4 training days was 87% [81%, 93%]. On average, accuracy improved across the 4 training days (Fig. 3*A*), but the difference was not significant, as measured by a 4-way repeated-measures ANOVA (*F*_(3,42)_ = 1.076, *p* = 0.370). The average accuracy during the scanning sessions was 69% [58%, 80%], with no significant difference between the two sessions (*t*_(15)_ = 0.786, *p* = 0.444, d_z_ = 0.20). Therefore, we can be reasonably confident that participants paid attention to the videos during observational training and scanning sessions.

Posttraining, sequence initiation time for the trained sequences (M = 600 ms [526 ms, 674 ms]) was significantly faster than for the untrained sequences (M = 684 ms [612, 756]) (*t*_(14)_ = 2.238, *p* = 0.042, d_z_ = 0.56, B_0_ = −84 ms [−165, −4]; Fig. 3*B*). Execution time for the trained sequences (M = 1338 ms [1215 ms, 1461 ms]) was significantly faster than for the untrained sequences (M = 1464 ms [1365, 1562]) (*t*_(14)_ = 3.495, *p* = 0.004, d_z_ = 0.87, B_0_ = −115 ms [−185, −45]; Fig. 3*C*). Therefore, effects sizes for our primary behavioral measures of observational learning (initiation and execution time) are typically considered medium and large according to Cohen's benchmarks (Cohen, 1992). Error rate did not differ between the two conditions (posttrained M = 12% [7, 18]; post-untrained M = 13%, [9, 18]), *t*_(14)_ = 0.319, *p* = 0.754, d_z_ = 0.08, B_0_ = −0.6% [−5, 4]; Fig. 3*D*).

### fMRI data

#### Univariate analyses

##### Brain regions engaged in action observation.

**Figure 4.**
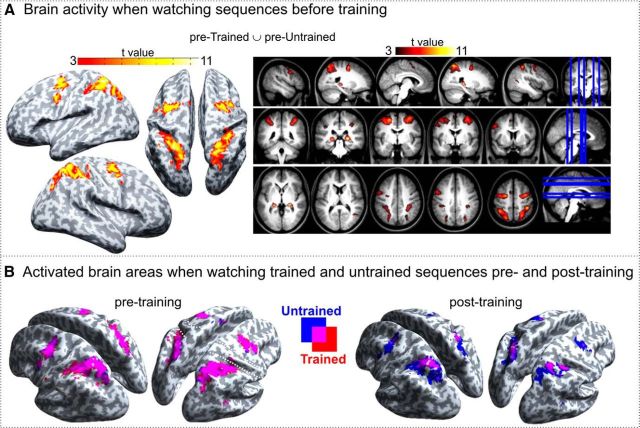
Univariate results for observing action sequences in general and across trained and untrained sequences. ***A***, Activated brain regions when watching sequences before the training (pretrained ∪ pre-untrained > implicit baseline). Statistical maps are overlaid on inflated standard MNI cortical surface (SPM12) and a group-average T1-weighted image in MNI template space. Maps are thresholded at a single voxel level (*p* < 0.001 uncorrected, *k* = 10), showing only clusters with cluster FWE-corrected significance at *p* < 0.05. ***B***, Brain activity maps of trained (red) and untrained (blue) conditions pretraining and posttraining. Maps are thresholded at a single voxel level *p* < 0.001 (uncorrected), *k* = 10.

To identify brain regions engaged when watching sequences in general, a group average contrast of pretrained ∪ pre-untrained > implicit baseline was assessed. The brain regions that emerged from this contrast included bilateral superior and inferior parietal lobules, intraparietal sulci, dorsal premotor cortices (including supplementary motor area), hippocampi, and left ventral premotor cortex (PMv). A list of the major peaks of activated clusters is given in Table 1 and all activated areas visualized in Figure 4*A*. Apart from no activation in the primary motor areas, the other activated areas closely matched those reported in the prior physical training study on which the current study was based (Wiestler and Diedrichsen, 2013). The activated bilateral frontoparietal regions largely correspond to the action observation network identified in previous studies (Cross et al., 2009; Caspers et al., 2010; Molenberghs et al., 2012; Kirsch and Cross, 2015). Brain activity maps of the trained and untrained conditions pretraining and posttraining are visualized in Figure 4*B*.

**Table 1.** Activated brain regions when watching sequences before training (pre-trained ∪ pre-untrained > implicit baseline)

Anatomical location	Cytoarchitectonic location	Peak MNI coordinates	Cluster level		Voxel level	
		*x y z*	voxels	*P*_FWE-corr_	*P*_FWE-corr_	*t*_15_
L Superior parietal lobule	7PC	−30 −56 60	1845	< 0.001	0.001	11.48
L Superior parietal lobule	7A	−20 −70 56			0.010	9.21
L Intraparietal sulcus	hIP3	−36 −50 54			0.014	8.98
R Inferior parietal lobule	Area 2	40 −40 54	1702	< 0.001	0.002	10.61
R Superior parietal lobule	7A	24 −64 58			0.003	10.19
R Intraparietal sulcus	hIP3	26 −56 58			0.010	9.17
L PMd, Superior frontal gyrus		−20 −6 54	1261	< 0.010	0.008	9.38
L PMv, Precentral gyrus		−32 −8 48			0.051	7.90
L PMv, Precentral gyrus	Area 44	−48 4 38			0.117	7.19
R PMd, Middle frontal gyrus		34 −4 54	759	< 0.001	0.013	9.00
R Hippocampus		22 −32 0	179	0.010	0.000	12.50
L Hippocampus		−22 −34 0	123	0.046	0.002	10.58

Results are thresholded at a single voxel level, *p* < 0.001, *k* = 10 voxels. Only clusters with cluster FWE-corrected significance at *p* < 0.05 are shown and up to three local maxima when a cluster has multiple peaks more than 8 mm apart.

##### Brain regions sensitive to observational training.

**Figure 5.**
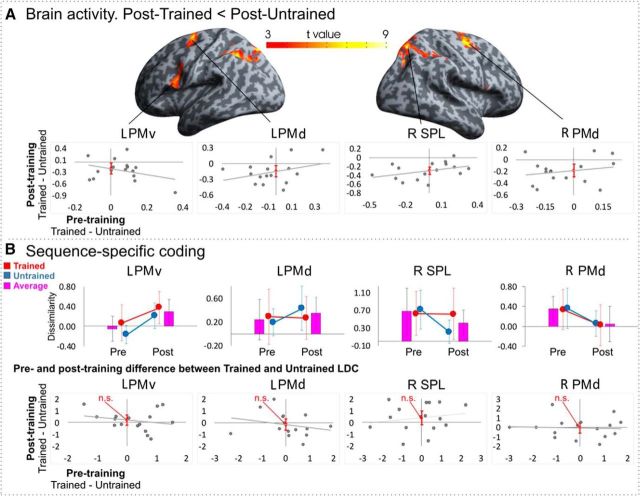
Univariate and MVPA ROI results. ***A***, Univariate results of posttraining difference between trained and untrained conditions corrected for pretraining difference. Maps are thresholded at a single voxel level (*p* < 0.001 uncorrected, *k* = 10, showing only clusters with cluster FWE-corrected significance at *p* < 0.05). Plots illustrate pretraining and posttraining difference in β weights between trained and untrained conditions in the four significant regions selected for further ROI analyses. Error bars represent 95% confidence interval of the intercept. ***B***, Top, MVPA results of sequence-specific coding pretraining and posttraining in the four ROIs showing dissimilarity estimate (average LDC value) between the sequences within the trained and untrained conditions and across both conditions on average. Bottom, Pretraining and posttraining difference between trained and untrained LDC. Error bars represent 95% confidence interval of the intercept. n.s., Nonsignificant.

The post-untrained > posttrained contrast revealed clusters in the right superior parietal lobule (SPL, extending across right precuneus and left superior and inferior parietal lobules), bilateral dorsal premotor cortices, and left PMv (Fig. 5*A*, Table 2). Therefore, after the 4 days of observational training, these brain regions showed decreased brain activity when watching trained compared with untrained sequences, which is consistent with prior physical training effects using the same sequences (Wiestler and Diedrichsen, 2013) and observational learning studies using similar sequence learning paradigms (Sakreida et al., 2018). No regions with higher activity for trained compared with untrained were found (Wiestler and Diedrichsen, 2013).

**Table 2.** Brain regions showing lower activity for trained compared with untrained sequences posttraining

Anatomical location	Cytoarchitectonic location	Peak MNI coordinates	Cluster level		Voxel level	
		x y z	Voxels	*P*_FWE-corr_	*P*_FWE-corr_	*t*_14_
**R SPL**	**7A**	**22 −68 56**	**1710**	**<0.001**	**0.007**	**9.43**
R Precuneus		10 −58 48			0.068	7.86
L Intraparietal sulcus	hIP3	−28 −50 40			0.210	7.16
**R PMd, Superior frontal gyrus**		**30 −4 58**	**610**	**<0.001**	**0.049**	**8.07**
R PMd, Precentral gyrus		28 −6 50			0.066	7.88
R PMd, Posterior-medial frontal cortex		16 −4 62			0.979	5.09
**L PMv, Inferior frontal gyrus (opercularis)**	**Area 44**	**−44 2 24**	**372**	**<0.001**	**0.708**	**5.94**
L PMv, Inferior frontal gyrus (opercularis)	Area 44	−56 8 10			0.891	5.50
L PMv, Precentral gyrus	Area 44	−50 6 20			0.958	5.24
**L PMd, Superior frontal gyrus**		**−24 −4 60**	**321**	**<0.001**	**0.044**	**8.14**
L PMd, Middle frontal gyrus		−24 −6 50			0.814	5.71
L PMd, Middle frontal gyrus		−12 −4 58			0.994	4.88

Results are thresholded at a single voxel level, *p* < 0.001, k = 10 voxels. Only clusters with cluster FWE-corrected significance at *p* < 0.05 are shown and up to three local maxima when a cluster has multiple peaks more than 8 mm apart. Highest peaks within each cluster selected for ROI analyses are shown in bold.

The opposite contrast (posttrained > post-untrained) did not result in any significant clusters.

We hypothesized that brain regions that show decreased activity following training would also show distinctive patterns of activity for different sequences in general, as well as for trained compared with untrained sequences (Fig. 1*E*). To investigate this hypothesis, we performed a MVPA on the four ROIs that showed a reduced BOLD response for trained compared with untrained sequences. In addition, we performed an exploratory MVPA using a searchlight approach across the whole volume.

#### MVPA

##### Sequence-specific representations of observed actions.

LDC analyses were used to test whether brain regions hold sequence-specific information following the observation of action sequences and whether the coding of such information is more distinct for trained compared with untrained sequences. The average dissimilarity (LDC value) of activity patterns between the four sequences within each condition was used as a measure of sequence-specific representations.

##### ROI approach.

We evaluated four ROIs that were sensitive to observational practice (Fig. 5*A*, Table 2): left ventral and dorsal portions of premotor cortex, right SPL, and right dorsal premotor cortex (PMd). Each ROI contained an average of 325 voxels (SD = 48.83). On average, across trained and untrained conditions posttraining, sequence-specific activity patterns were found in the left ventral and dorsal portions of premotor cortex and right SPL (Fig. 5*B*, Table 3). More specifically, sequence-specific activity patterns were found in left ventral premotor and left dorsal premotor cortices only at posttraining; in the right SPL both at pretraining and posttraining; and in the right PMd only at pretraining. The effect sizes were medium to large in magnitude according to Cohen's benchmark criteria (Cohen, 1992), ranging from Cohen d_z_ = 0.65 to 0.77 (Table 3). These results show that parts of frontoparietal cortex that show sensitivity to observational learning, as measured by changes in average activity, also show distinctive patterns of activity as a function of observed key press sequences.

**Table 3.** Sequence-specific coding in ROIs

ROI	Mean LDC, one-sample, one-tailed *t* test				Posttrained vs post-untrained
L PMv					
Pre:	−0.05 [−0.26, 0.16]	n.s.		d_z_ = 0.11	B_0_ = 0.22 [−0.26, 0.70], n.s., d_z_ = 0.25
Post:	0.29 [0.10, 0.49]	t_15_ = 2.59	*p* = 0.01	d_z_ = 0.65	
L PMd					
Pre:	0.24 [−0.04, 0.52]	n.s.		d_z_ = 0.38	B_0_ = −0.14 [−0.66, 0.39], n.s., d_z_ = 0.14
Post:	0.35 [0.12, 0.58]	t_15_ = 2.69	*p* = 0.008	d_z_ = 0.67	
R SPL					
Pre:	0.68 [0.24, 1.11]	t_15_ = 2.7	*p* = 0.008	d_z_ = 0.68	B_0_ = 0.41 [−0.22, 1.05], n.s., d_z_ = 0.35
Post:	0.42 [0.18, 0.65]	t_15_ = 3.08	*p* = 0.004	d_z_ = 0.77	
R PMd					
Pre:	0.35 [0.14, 0.56]	t_15_ = 2.91	*p* = 0.005	d_z_ = 0.73	B_0_ = −0.04 [−0.64, 0.57], n.s., d_z_ = 0.03
Post:	0.04 [−0.25, 0.33]	n.s.		d_z_ = 0.06	

n.s., Nonsignificant.

In the same ROIs, there was only suggestive evidence that sequence-specific representational dissimilarity was different between trained and untrained sequences at the post test (Fig. 5*B*, Table 3). In two ROIs, there was a trend toward sequence-specific representations in frontoparietal cortex showing training-specific effects. In these two ROIs, the training-specific effects at the posttraining scan (trained > untrained) were small to medium in size (Cohen's d_z_ = 0.25 for left PMv and 0.35 for superior parietal cortex). However, none of the ROIs showed a significant effect of training. Therefore, 4d of observational training produced relatively weak evidence that regions of frontoparietal cortex develop more distinctive sequence-specific patterns of activity when observing trained compared with untrained sequences.

To complement these targeted tests of our primary hypotheses, we also performed a 4 (region; L PMv, L PMd, R SPL, R PMd) × 2 (scan session: pretest, posttest) × 2 (training: trained, untrained) ANOVA on LDC values (Table 4). This analysis revealed a main effect of region *F*_(3,45)_ = 4.373, *p* = 0.009, η_*p*_^2^ = 0.226 and a region * scan session interaction *F*_(3,45)_ = 2.904, *p* = 0.045, η_*p*_^2^ = 0.162. For both effects, the effect size (partial η squared, η_*p*_^2^) is conventionally considered large. All other effects did not approach significance and effect sizes were close to zero or small.

**Table 4.** Sequence-specific coding (LDC values) analyzed by region, session, and training type

Three-factor ANOVA	df	*F*	*p*	η_p_^2^
Region × scan session × training				
Region	3, 45	4.373	0.009	0.226
Scan session	1, 15	0.027	0.871	0.002
Training	1, 15	0.616	0.445	0.039
Region × scan session	3, 45	2.904	0.045	0.162
Region × training	3, 45	0.429	0.733	0.028
Scan session × training	1, 15	0.022	0.885	0.001
Region × scan session × training	3, 45	0.973	0.414	0.061
Two-factor ANOVA by region				
L PMv, scan session × training				
Scan session	1, 15	3.916	0.066	0.207
Training	1, 15	2.367	0.145	0.136
Scan session × training	1, 15	0.046	0.833	0.003
L PMd, session × training				
Scan session	1, 15	0.189	0.670	0.012
Training	1, 15	0.050	0.827	0.003
Scan session × training	1, 15	0.469	0.504	0.030
R SPL, session × training				
Scan session	1, 15	0.707	0.414	0.045
Training	1, 15	0.429	0.522	0.028
Scan session × training	1, 15	1.606	0.224	0.097
R PMd, session × training				
Scan session	1, 15	3.247	0.092	0.178
Training	1, 15	0.021	0.887	0.001
Scan session × training	1, 15	<0.001	0.987	<0.001

The structure of the three-factor ANOVA is a 4 (region: L PMv, L PMd, R SPL, R PMd) × 2 (scan session: pretest, posttest) × 2 (training: trained and untrained) repeated-measures ANOVA. The structure of the two-factor ANOVA is a 2 (scan session: pretest, posttest) × 2 (training: trained and untrained) repeated-measures ANOVA.

To investigate the region * session interaction, we split the data by region and performed four further 2 (scan session: pretest, posttest) × 2 (training: trained, untrained) ANOVAs. Two clear patterns emerged. For left PMv, there was a large main effect of scan session (*F*_(1,15)_ = 3.916, *p* = 0.066, η_*p*_^2^ = 0.207), such that dissimilarity values were higher at posttest than at pretest (Fig. 5*B*). Right PMd also showed a large main effect of scan session (*F*_(1,15)_ = 3.247, *p* = 0.092, η_*p*_^2^ = 0.178), such that dissimilarity values were lower at posttest than at pretest (Fig. 5*B*). Although both of these main effects of scan session only approach a conventional significance level using null hypothesis significance testing (i.e., *p* < 0.05), the presence of large effect sizes in these regions combined with much smaller effects in other regions (Table 4) demonstrates that these two regions were driving the region * scan session interaction in the omnibus three-factor ANOVA. Finally, the main effect of region reflects higher dissimilarity values on average across scan session and training type in right SPL than the other ROIs. In sum, out of the four ROIs, left PMv shows the strongest evidence for a pretest to posttest increase in dissimilarity values between sequences with the effect of training generalizing from trained to untrained sequences (Fig. 5*B*).

##### Searchlight approach.

**Figure 6.**
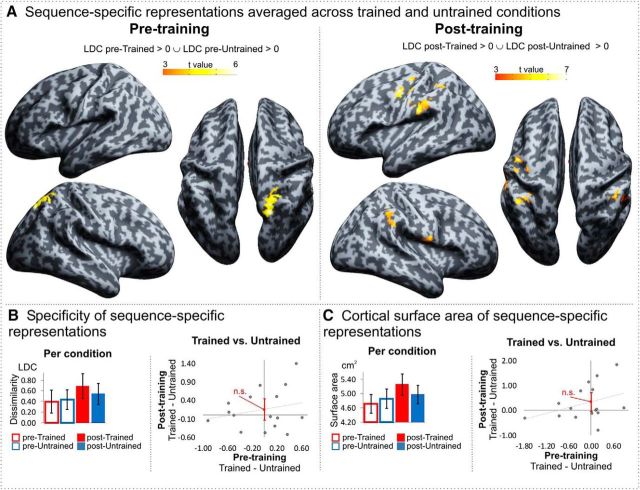
MVPA searchlight results. ***A***, Pretraining and posttraining sequence-specific representations. Maps are thresholded at a single voxel level (*p* < 0.001 uncorrected, *k* = 10), showing only clusters with cluster FWE-corrected significance at *p* < 0.05. ***B***, ***C***, Specificity (the average LDC measure) of sequence-specific representations and the cortical surface area coding sequence-specific representations averaged over all involved cortical regions per condition (left; error bars represent within-subject 95% confidence interval; *p < 0.05) and pretraining and posttraining difference (right; error bars represent 95% confidence interval of the intercept. n.s., Nonsignificant at *p* < 0.05).

A whole-volume exploratory surface-based searchlight analysis revealed pretraining (averaged across pretrained and pre-untrained conditions) sequence-specific activity patterns in the right anterior intraparietal sulcus and posterior SPL (Fig. 6*A*, left, Table 5). In addition, posttraining (averaged across posttrained and post-untrained conditions), sequence-specific activity patterns were found in bilateral supramarginal gyri, anterior intraparietal sulci (homologous to macaque AIP; Culham et al., 2006), left anterior SPL, left primary motor and somatosensory cortices, and right parietal operculum (Fig. 6*A*, right, Table 5).

**Table 5.** Brain regions showing sequence-specific coding for trained ∪ untrained conditions pretraining and posttraining

Anatomical location	Cytoarchitectonic location	Peak MNI coordinates	Cluster level		Voxel level		Average LDC
		*x y z*	voxels	*P*_FWE-corr_	*P*_FWE-corr_	*t*_15_	
Pretraining							
R IPS	hIP3	22 −62 58	453	< 0.001	0.543	5.88	0.95
R SPL	7A	20 −68 50			0.590	5.79	1.02
R SPL		20 −56 48			0.914	5.04	0.52
Posttraining							
L Supramarginal gyrus	PFop	−56 −26 22	269	0.001	0.377	6.32	0.82
L Supramarginal gyrus	PFt	−56 −24 32			0.949	4.96	0.74
L Supramarginal gyrus	PFt	−66 −26 38			0.995	4.53	0.30
L M1, Precentral gyrus	4a	−50 −10 42	157	0.020	0.170	7.04	0.77
L M1, Postcentral gyrus	4p	−42 −8 34			0.849	5.29	0.32
L S1, Postcentral gyrus	3b	−46 −16 48			0.994	4.57	0.88
R IPS	hIP2	48 −38 42	145	0.029	0.971	4.83	0.96
R Supramarginal gyrus	PF	58 −40 30			0.997	4.46	0.74
R IPL	Area 2	48 −36 52			1.000	4.24	0.71
L IPS	hIP2	−46 −48 54	143	0.030	0.907	5.12	0.92
L SPL	5L	−32 −42 46			0.970	4.48	0.55
R Parietal operculum	OP4	58 −8 12	134	0.039	0.874	5.22	0.70

Results thresholded at a single voxel level, *p* < 0.001, *k* = 10 voxels. Only clusters with cluster FWE-corrected significance at *p* < 0.05 are shown and up to three local maxima when a cluster has multiple peaks more than 8 mm apart.

M1, Primary motor cortex; S1, primary somatosensory cortex; IPL, inferior parietal lobule; IPS, intraparietal sulcus; SPL, superior parietal lobule.

Similar to the ROI analyses, there was only suggestive evidence that sequence-specific activity patterns become more distinct when watching trained compared with untrained sequences following observational training. For example, at a cluster FWE-corrected threshold of *p* < 0.05, no brain regions showed sequence-specific and training-specific patterns of activity. In addition, when comparing the sequence-specific activity patterns globally, averaging over all involved cortical regions, there was only suggestive evidence for more distinct and more widespread sequence-specific coding following practice. Specifically, the average LDC measure of the posttrained sequences was higher than of the post-untrained sequences; however, the difference was not significant (*t*_(14)_ = 1.128, *p* = 0.278, d_z_ = 0.28, B_0_ = 0.155 [−0.139, 0.449]; Fig. 6*B*). Similarly, the average cortical surface area coding sequence-specific representations of the posttrained sequences was larger than of the post-untrained, but the difference was not significant (*t*_(14)_ = 1.935, *p* = 0.073, d_z_ = 0.48, B_0_ = 0.34 cm^2^ [−0.035, 0.715]; Fig. 6*C*).

## Discussion

The neural changes that underpin how visual signals are mapped onto motor circuits when we learn by observation have remained largely unclear. Here, we show that observed action sequences are modeled by distinct patterns of activity in frontoparietal cortex and that such representations largely generalize to very similar, but untrained, sequences. These findings advance our understanding of what is modeled during observational learning (sequence-specific information), as well as how it is modeled (reorganization of frontoparietal cortex is similar to that previously shown following physical practice). Therefore, on a more fine-grained neural level than demonstrated previously, we show how the representational structure of frontoparietal cortex maps visual information onto motor circuits in order to enhance motor performance.

### Sequence-specific activation patterns in frontoparietal brain regions during the observation of action sequences

Prior work has shown that physically practicing key press sequences leads to both reduced engagement of frontoparietal cortex, as well as more distinct patterns of sequence-specific activity in these same brain regions (Wiestler and Diedrichsen, 2013). Here, we show that observation of action sequences also leads to a similar functional reorganization of frontoparietal cortex. Right SPL, left PMd and left PMv showed a reduction in engagement after visual training of these sequences (Vogt et al., 2007; Higuchi et al., 2012; Sakreida et al., 2018), as well as sequence-specific patterns of activity. The results show close correspondence to prior work on physical practice (Wiestler and Diedrichsen, 2013) by demonstrating that similar regions that distinguish between physically practiced sequences also show sequence-specific patterns when sequences are trained via observation. Moreover, the searchlight analysis showed that premotor and parietal cortices, rather than primary motor cortex, showed sequence-specific representations. Therefore, similar levels of the motor system hierarchy (Abrahamse et al., 2013; Diedrichsen and Kornysheva, 2015) appear to be modified following physical and observational exposure to action sequences. As such, our data suggest that patterns of activity in frontoparietal cortices represent observed action sequences in a manner similar to physically performed action sequences.

It is important to acknowledge that we did not include a physical practice condition in our experiment and are therefore unable to make direct comparisons between observed and executed action sequences. We specifically chose to include only observational practice and untrained conditions in the current study for several reasons. First, this approach enables us to boost the power of our primary analysis, which focused on observed sequences. Second, including a physical training condition makes it more difficult to disentangle which learning-related changes are due to observational experience per se and which might be at least partially attributable to carryover effects from physical practice. Third, by exclusively focusing on observational learning, we could still benefit from comparisons to previously published research that featured physical practice of the identical sequences (Wiestler and Diedrichsen, 2013). Finally, by using a functional ROI approach, we are able to provide a functional characterization of the regions under investigation. That is, the regions studied in our experiment show a reduced magnitude of response following observational practice, just as similar frontoparietal brain regions do following physical practice (Wiestler and Diedrichsen, 2013). Although less conclusive than direct comparisons between conditions, the functional definition of ROIs does support the view that similar portions of frontoparietal cortex reorganize in a similar manner following observational and physical practice.

The results update our understanding of the role of frontoparietal cortex in shared representations between action and perception in general (Gentsch et al., 2016), as well as our understanding of the features modeled during observational learning (Blandin et al., 1999; Hodges et al., 2007; Boutin et al., 2010). Prior work has shown that the observation and performance of action share cognitive and neural mechanisms (Prinz, 1997; Giese and Rizzolatti, 2015; Gentsch et al., 2016), which span different levels of the motor hierarchy (e.g., intentions, goals, motor commands; Grafton and Hamilton, 2007). In the present study, sequences were similar to each other at all levels of the motor hierarchy (intentions, goals, motor commands) and differed only in the sequential order of key presses. Despite the close similarity between the individual actions, we found sequence-specific representations in different parts of frontoparietal cortex. This result deepens understanding of what is shared between perception and production of action (de Vignemont and Haggard, 2008), which may not have been possible using conventional univariate analyses. Indeed, analyses that average activity across voxels in a region may have been unable to demonstrate any differentiation between observed action sequences (Frey and Gerry, 2006; Sakreida et al., 2018), resulting in a conclusion that observed sequences are represented in frontoparietal cortex in an indistinguishable and relatively coarse manner (e.g., five key presses in any order). Instead, by analyzing the pattern of activity across voxels, we show that, rather than observed sequences being represented on a relatively coarse scale, they are discriminable at an individual sequence level in frontoparietal cortex. This finding thus demonstrates how motor circuit involvement in perception of action sequences maintains high fidelity to physical performance.

### Neural plasticity following observational practice

Behavioral data show that observational training leads to faster initiation and movement times for trained compared with untrained sequences and decreases in neural activity within frontoparietal cortex, which mirrors results from physically practicing identical sequences (Wiestler and Diedrichsen, 2013). Therefore, in terms of averaged activity, similar neural efficiency or redundancy gains were seen following observational practice as physical practice (Wiestler and Diedrichsen, 2013; Higuchi et al., 2012). In addition, we show similar evidence of neural generalization following training: sequence-specific representations were measurable when observing trained and untrained sequences after 4 d of training, which replicates prior physical training effects (Wiestler and Diedrichsen, 2013) and is consistent with our behavioral data. These data show clear evidence of generalization of learning from trained to untrained sequences. Given that these sequences were visually and motorically very similar to each other and many of the trained sequences had similar finger transitions to the untrained sequences (Wiestler and Diedrichsen, 2013), it was expected that generalization would occur. Indeed, it is likely that transitions between key presses are “chunked” during learning and therefore benefit performance when those transitions are present in the untrained sequences (Wymbs et al., 2012). For example, many transitions between finger movements were shared between trained and untrained sequences, which means that practicing a transition on trained sequences would help one perform other sequences that also include that particular transition, even if the overall sequence is different.

Wiestler and Diedrichsen (2013) also showed that physical practice leads to more distinct sequence-specific representations for trained compared with untrained sequences in frontoparietal cortex. The current study only shows suggestive evidence that following observational learning sequence-specific representations in frontoparietal cortex are more distinctive for trained compared with untrained sequences. For example, in our ROI approach, at posttest, training-specific effects of MVPA were relatively small (Cohen's d 0.25 and 0.35) and did not reach a predefined statistical threshold of *p* < 0.05. Further, in our ROI analysis, only left PMv showed evidence for a pretest to posttest increase in dissimilarity values for observed sequences, which is consistent with observational training leading to an increase in the representational distinctiveness of observed action sequences. By using a functional ROI approach, we are able to make inferences regarding representational similarity changes in regions that show sensitivity to observational learning as measured by magnitude changes. To this end, we are able to infer that of the brain regions that show robust univariate sensitivity to observational learning, left PMv shows the strongest evidence for increased representational reorganization as a function of observed sequence learning.

Our conclusions are not solely based on the functional ROI approach, however, as we also performed a whole-volume searchlight analysis, which does not involve restricting the search volume to functional areas of interest. Although evidence in support of training-related differences in representational distinctiveness was relatively weak, the whole-volume searchlight approach also suggested that frontoparietal cortex develops training-specific and sequence-specific representations, which cover a greater proportion of cortex following training. Although more robust than what we report here, it is worth noting that the effects of physical practice in prior work were also rather subtle (Wiestler and Diedrichsen, 2013). In a global analysis that averaged activity in frontal and parietal areas, the effect of physical training corresponded to a 4% increase. Further, in a whole map analysis, only left supplemental motor area (SMA)/pre-SMA showed a reliable effect for trained compared with untrained sequences. Given that the behavioral effect of observational learning is smaller than physical learning, it is possible that observational learning results in more distinctive sequence-specific patterns of activity, but the effect sizes are smaller than physical practice and therefore harder to detect. Given the similarity in behavioral training effects between physical and observational learning of sequences, as well as the similarity in magnitude-based measures of neural activity in frontoparietal cortex, we suggest that this interpretation is likely. Alternatively, it is possible that observational learning does not lead to modified patterns of activity that are sequence and training specific in a manner similar to physical learning. Only future research will be able to confirm or deny these possibilities.

Together, these findings provide a more general insight into the functional reorganization of frontoparietal cortex following observational learning. If only univariate results are considered, then reduced engagement of frontoparietal cortex is consistent with greater efficiency in neural function: reduced and less widespread neural engagement is associated with improved physical performance (Steele and Penhune, 2010). However, by unpacking the representational structure of frontoparietal cortex in a sequence-specific manner, we are able to show that frontoparietal cortex develops a richer and more widespread representation of observed action sequences, which largely generalizes to untrained sequences. Previous research based on averaging activity across voxels has fueled much debate about the relative contribution of increased or decreased engagement of the motor system in learning (Steele and Penhune, 2010; Dayan and Cohen, 2011; Gardner, Aglinskas and Cross, 2017). Extending this work, we emphasize here that unlocking the code that is hidden within averaged activity can provide an altogether different understanding of brain organization (Norman et al., 2006; Kriegeskorte et al., 2008). Moreover, the results highlight the value of using representational similarity analyses in the context of learning to understand plasticity, which few studies have focused on to date (Kriegeskorte and Kievit, 2013).

### Limitations

In the present study, all eight sequences (four to-be-trained and four untrained) were physically performed before the 4 d of observational training. Therefore, the posttraining performance improvement, at least partly, could be driven by the consolidation of physical performance (Censor et al., 2012). Although some contribution of physical practice is possible, there was considerably more observational practice (over 100 observations per sequence versus five executions). Moreover, trained and to-be-untrained sequences were all physically practiced before the first scan, so any comparisons between trained and untrained sequences were matched for physical practice. For these reasons, we do not think that physical practice had a substantial influence on training-specific effects.

Differences between the current results and those obtained previously from physical practice may result from different dose-response relationships. Although the behavioral and univariate effects of observational training were quite large, the potency of observational practice is likely to be less than physical practice. Therefore, if we had provided sufficient training through observational practice to match the behavioral training gains following physical practice, then an even closer set of results may emerge between observational and physical training. Further, we also acknowledge that participants in the current study were not told to intentionally learn the observed action sequences; instead, participants were told to detect errors. Therefore, it is possible that the training effects would be larger if participants were given a clear intention to learn. Nonetheless, it remains clear that unintentional learning leads to the type of cognitive and neural reorganization, which has been outlined in the present study. Future work investigating the effect of intentionality in learning using representational similarity analyses would be of interest.

A further limitation concerns the extent to which the reported effects are specific to learning action sequences or learning a more general form of sequence information that is not tied to action sequences per se. To preserve the power of our design, we did not include a separate sequence learning condition that did not include action sequences (such as symbolically cued sequences). Therefore, we are unable to directly compare different types of sequence learning. However, prior studies have shown that behavioral performance measures dissociate between observational practice based on symbolic and action cues (Bird et al., 2005). Therefore, it is likely that some of the effects reported will be related to action-specific features, whereas others are likely to be related to more general spatial features of the stimuli. As a consequence, and consistent with recent proposals in neuroscience research more generally (Spunt and Adolphs, 2017), an important and interesting line of future research would be to identify how domain-general and domain-specific systems make independent, as well as interactive, contributions to observational learning.
